# Fine-mapping and transcriptome analysis of a candidate gene controlling plant height in *Brassica napus* L.

**DOI:** 10.1186/s13068-020-01687-y

**Published:** 2020-03-10

**Authors:** Xiaodong Wang, Ming Zheng, Hongfang Liu, Liang Zhang, Feng Chen, Wei Zhang, Shihang Fan, Menlu Peng, Maolong Hu, Hanzhong Wang, Jiefu Zhang, Wei Hua

**Affiliations:** 1 Oil Crops Research Institute of the Chinese Academy of Agricultural Sciences, Key Laboratory of Biology and Genetic Improvement of Oil Crops, Ministry of Agriculture and Rural Affairs, Wuhan, China; 2 Provincial Key Laboratory of Agrobiology, Institute of Industrial Crops, Jiangsu Academy of Agricultural Sciences, Key Laboratory of Cotton and Rapeseed, Ministry of Agriculture and Rural Affairs, Nanjing, China

**Keywords:** *Brassica napus* L., Plant height, Quantitative trait loci sequencing, Fine-mapping, Transcriptome analysis, Candidate gene, Molecular marker

## Abstract

**Background:**

*Brassica napus* provides approximately 13–16% of global vegetable oil for human consumption and biodiesel production. Plant height (PH) is a key trait that affects plant architecture, seed yield and harvest index. However, the genetic mechanism of PH in *B. napus* is poorly understood.

**Results:**

A dwarf mutant *df59* was isolated from a large-scale screening of an ethyl methanesulphonate-mutagenized rapeseed variety Ningyou 18. A genetic analysis showed that the dwarfism phenotype was controlled by one semi-dominant gene, which was mapped on C9 chromosome by quantitative trait loci sequencing analysis and designated as *BnaDwf.C9*. To fine-map *BnaDwf.C9*, two F_2_ populations were constructed from crosses between conventional rapeseed cultivars (Zhongshuang 11 and Holly) and *df59*. *BnaDwf.C9* was fine-mapped to the region between single-nucleotide polymorphism (SNP) markers M14 and M4, corresponding to a 120.87-kb interval of the *B. napus* ‘Darmor-*bzh*’ genome. Within this interval, seven, eight and nine annotated or predicted genes were identified in “Darmor-*bzh*”, “Ningyou 7” and “Zhongshuang 11” reference genomes, respectively. In addition, a comparative transcriptome analysis was performed using stem tips from Ningyou 18 and *df59* at the stem elongation stage. In total, 3995 differentially expressed genes (DEGs) were identified. Among them, 118 DEGs were clustered in plant hormone-related signal transduction pathways, including 81 DEGs were enriched in auxin signal transduction. Combining the results of fine-mapping and transcriptome analyses, *BnaC09g20450D* was considered a candidate gene for *BnaDwf.C9*, which contains a SNP that co-segregated in 4746 individuals. Finally, a PCR-based marker was developed based on the SNP in *BnaC09g20450D*.

**Conclusions:**

The combination of quantitative trait loci sequencing, fine-mapping and genome-wide transcriptomic analysis revealed one candidate gene located within the confidence interval of 120.87-kb region. This study provides a new genetic resource for semi-dwarf breeding and new insights into understanding the genetic architecture of PH in *B. napus*.

## Background

Rapeseed (*Brassica napus*, AACC, 2*n* = 38) is not only an important oilseed crop worldwide, but also an emerging biofuel crop. Rapeseed oil is an ideal vegetable oil for human consumption, because it contains ~ 65% oleic, ~ 20% linoleic, ~ 9% linolenic and a very low level of stearic acid [1]. Vegetable oils are triglyceride sources for biodiesel production. In Europe, biodiesel has been produced mainly from rapeseed oil [2]. In addition, rape straw is an abundant lignocellulosic material for the production of liquid biofuel, particularly ethanol. In China, the cultivated area of rapeseed is ~ 67 million hectares, with an annual seed yield of ~ 4.5 million tons every year [3], and the collectable production of rape straw was 38.17 million tons in 2013 [4]. Plant height (PH) is a key trait that affects the plant architecture, seed yield, dry weight and harvest index [5]. Moreover, PH is the major contributor to lodging tolerance, a serious abiotic stress during rapeseed production. Lodging makes *B. napus* unsuitable for mechanical harvesting and causes dramatic decreases in yield and seed quality [6]. Therefore, it is important to understand the genetic bases of PH to breed new cultivars with an ideal plant architecture and to maximize *B. napus*’ economic benefits as an oil and bioenergy crop.

Plant growth and development consist of very precise and complicated procedures. The molecular mechanisms regulating PH in the model plant *Arabidopsis thaliana* and rice (*Oryza sativa*) have been well recorded, and most of the genes are involved in phytohormone-related pathways [7–11]. Gibberellic acid (GA) is an essential endogenous regulator of PH. For example, the rice ‘green revolution’ gene *sd1* encodes GA20ox2, which is an oxidase enzyme involved in the biosynthesis of GA [12]. The wheat ‘green revolution’ gene *Rht*-*B1/Rht*-*D1* encodes a DELLA protein that acts as a negative regulator in the GA-signaling pathway [13]. Auxins play pivotal functions in developmental processes, because they are involved in controlling virtually every aspect of plant biology [14]. Indole-3-acetic acid (IAA) is the key auxin in most plants, and it is mainly biosynthesized from tryptophan (Trp) through a Trp-dependent pathway [14, 15]. Loss-of-function mutations in IAA-related Trp-dependent biosynthetic genes (e.g., *TAA1* or *YUCCA*) can seriously affect PH [16, 17]. In the auxin-signaling pathway, transport inhibitor resistant1/auxin-signaling F-box (TIR1/AFB), auxin/indole acetic acid proteins (Aux/IAAs) and auxin response factors (ARFs) are three key factors regulating auxin-induced gene expression [18, 19]. At low auxin levels, the transcriptional repressor Aux/IAA proteins and the corepressor TOPLESS interact with ARF proteins, resulting in auxin-induced gene repression. At high auxin levels, auxin interacts with TIR1, leading to the formation of TIR1/AFB-Aux/IAA-ARF complexes, then ubiquitin-ligase (E3) ubiquitinates AUX/IAAs, forming the ubiquitin 26S proteasome responsible for the degradation of AUX/IAAs [16, 18, 20]. Free ARF proteins can activate the transcription of auxin-response genes. Mutations that occur in the genes involved in auxin signaling may result in typical dwarf phenotypes, such as *IAA7* in *B. napus* [21].

*Brassica napus* is a tetraploid species, which originated from a hybridization between *B. rapa* (AA, 2*n* = 20) and *B. oleracea* (CC, 2*n* = 18) around 7500 years ago [22]. In *B. napus*, many studies have focused on quantitative trait loci (QTLs) detection using linkage mapping or genome-wide association studies (GWASs), and hundreds of QTLs have been identified across all 19 chromosomes [23–31]. However, most QTLs explain a small percentage of the total phenotypic variance (PV) [23–31]. Only a few QTLs affecting PH have been fine-mapped and cloned in *B. napus*. Wang et al. obtained two dwarf mutants “*Bndwf1*” and “*Bndwf1/dcl1*”, and fine-mapped the associated QTLs on the A9 chromosome to a 152-kb interval and on the C5 chromosome to a 175-kb interval, respectively [32, 33]. Liu et al. mapped the semi-dominant gene *ds*-*1* (*BnaA06.RGA*) on the A6 chromosome. It encodes a DELLA protein that functions as a repressor in GA signaling [34]. A single proline (P)-to-leucine (L) change was identified in the VHYNP motif of a DELLA protein in ds-1 that leads to a gain-of-function and caused a dwarf phenotype [34]. Subsequently, the *DS*-*3* gene, which is syntenic to *ds*-*1* with a similar gene function but a weaker effect on PH, was mapped on the C7 chromosome [35]. In the auxin-signaling pathway, only two genes that encode Aux/IAA proteins were identified and functionally validated in *B. napus*, *BnaA3.IAA7* on the A3 chromosome and its homolog *BnaC05.IAA7* on the C5 chromosome [21, 36]. The molecular mechanisms regulating the PH of *B. napus* remain elusive, and elucidating the mechanism of a new dwarf gene has important scientific significance and applicable value.

The rapeseed ideotype is a semi-dwarf stature with a plant height of ~ 120–140 cm, narrow branch angles (< 30°), and a middle-long silique length [21, 37]. A dwarf mutant of *df59* with a PH of ~ 65 cm was obtained from ethyl methanesulphonate (EMS)-mutagenized Ningyou 18 (NY18). The *df59* is an excellent germplasm resource for semi-dwarf breeding, with the average PH of F_1_ hybrid lines from crosses between NY18 and *df59* being 126.75 ± 4.3 cm, which is in accordance with the ideotype criteria of *B. napus* [21, 37]. The aims of the present study were to: (1) fine-map the gene responsible for dwarf architecture in *df59* using QTL sequencing (QTL-seq) and map-based cloning strategies; (2) elucidate the patterns of gene expression between NY18 and *df59* using comparative transcriptomic analyses; and (3) develop a stable single-nucleotide polymorphism (SNP) marker tightly linked to the dwarf gene that could be used for marker-assisted selection. The present study provides a new gene source for the semi-dwarf breeding of new varieties, and the findings contribute to a better understanding of the molecular mechanisms underlying dwarfism.

## Results

### Phenotypic variation and genetic analysis of plant height

**Fig. 1:**
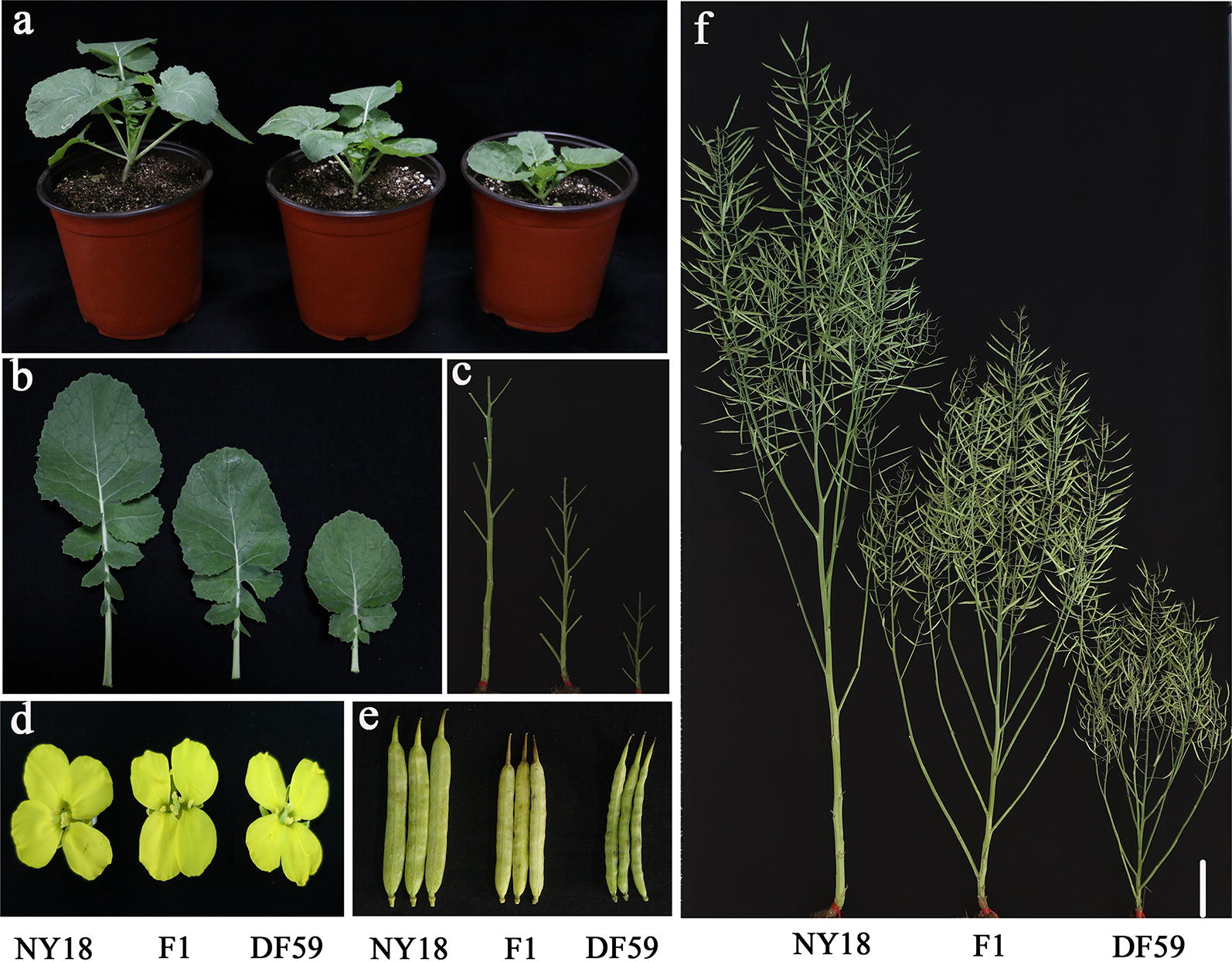
Phenotypic characterization of NY18, *df59*, and their F_1_ at different developmental stages. **a** Morphology of the NY18, *df59* and their F_1_ at the seedling stage; **b** comparison of leaf phenotypes at the seedling stage; **c** comparison of internode lengths at the mature stage; **d** comparison of petal phenotypes at the flowering stage; **e** comparison of silique-related traits at the mature stage; **f** comparison of plant heights and plant architecture at the mature stage. Scale bars = 10 cm

At the seedling stage, *df59* was already significantly shorter than NY18, with smaller leaves and shorter petioles (Fig. 1a, b). The internode length and PH were different between *df59* and NY18 at the mature stage (Fig. 1c), while the petals and siliques of *df59* were smaller than those of NY18 (Fig. 1d, e). At maturity, agronomic traits and seed yield-related traits were investigated in NY18, *df59* and the F_1_ of their cross (Table 1). Among the 15 traits, 12 were significantly higher in NY18 than in *df59* (Table 1), with the exception of the first effective branch number, pod number of main inflorescence and silique number per plant. The PH of the F_1_ was 126.75 ± 4.3 cm, which meets the ideotype requirement of ~ 120–140 cm and will be beneficial to high-density planting (Fig. 1f, Table 1). In addition, the seed yield per F_1_ plant was 28.4 ± 2.20 g, which was 84.02% of the NY18 yield (33.8 ± 2.51 g). The phenotypic values of seed oil content and seed fatty acid concentrations for the NY18, *df59* and the F_1_ are provided in Additional file 1: Table S1. F_1_ and NY18 had similar C16:0, C18:0, C18:2, C18:3, C20:1 and oil content, but had different C18:1 and C22:1 content. Theoretically, if the planting density of the F_1_ increased to 1.5 times, the seed yield would increase ~ 26% per unit of area compared with the normal planting density of NY18.

**Table 1:** Phenotypic values of agronomic traits, seed yield-related traits and root-related traits for NY18, *df59* and their F_1_

	Traits	NY18	*df59*	NY18 × *df59*	F1/NY18 (%)
Agronomic traits	Plant height (cm)	180.4 ± 3.6	65.2 ± 4.6	126.75 ± 4.3	70.26
	First effective branch height (cm)	59.4 ± 5.4	6.4 ± 2.6	20.25 ± 5.3	34.09
	First effective branch number	6.8 ± 0.4	10.4 ± 4.4	10.25 ± 2.6	150.73
	Length of main inflorescence (cm)	76.2 ± 3.4	40.2 ± 4.5	67.4 ± 10.8	88.45
	Pod number of main inflorescence	75.0 ± 10.9	61.6 ± 5.7	76.6 ± 5.0	102.13
	Biomass yield (g)	99.0 ± 3.7	46.9 ± 3.1	68.7 ± 2.7	69.41
	Internode length (cm)	8.04 ± 1.9	2.69 ± 0.6	5.53 ± 1.5	68.78
	Harvest index	0.34 ± 0.03	0.31 ± 0.05	0.41 ± 0.04	120.58
Seed yield-related traits	Seed yield (g)	33.8 ± 2.51	14.7 ± 1.44	28.4 ± 2.20	84.02
	Thousand seed weight (g)	5.23 ± 0.07	3.23 ± 0.06	3.56 ± 0.05	68.07
	Silique number per plant	245.8 ± 30.3	277.6 ± 60.2	303 ± 44.9	123.27
	Silique length (cm)	6.7 ± 0.3	5.5 ± 0.4	5.8 ± 0.2	86.57
	Silique breadth (mm)	7.31 ± 0.25	4.3 ± 0.35	5.43 ± 0.51	74.28
	Silique volume (cm^3^)	1.34 ± 0.12	0.44 ± 0.12	0.95 ± 0.12	70.89
	Seed number per silique	25.7 ± 2.2	14.6 ± 3.2	18.9 ± 2.1	73.54
Root-related traits	Total root length (cm)	37.9 ± 7.23	24.67 ± 5.04	31.0 ± 6.07	81.79
	Root surface area (cm^2^)	3.12 ± 0.55	2.25 ± 0.46	2.61 ± 0.47	83.65
	Root volume (cm^3^)	0.020 ± 0.004	0.0165 ± 0.003	0.018 ± 0.003	90.0
	Number of root tips	42.0 ± 8.3	27.8 ± 8.8	33.4 ± 7.1	79.52

Root-related traits, including total root length, root surface area, root volume and number of root tips, were measured for NY18, *df59* and their F_1_ at 10 days after germination (Additional file 2: Figure S1). The values of the four traits in *df59* were significantly less than in NY18, and the F_1_ had approximate mean values of the two parents, which were in accordance with the PH value (Table 1).

The 165 individuals of the F_2_ population from a cross between NY18 and *df59* (named NY–DF) observed contained 43 tall plants, 88 medium plants and 34 dwarf plants. A Chi-squared test indicated that the segregation pattern agreed with the Mendelian segregation ratio of 1:2:1 (χ^2^ = 1.719, *P* > 0.05). The mixed major-gene plus polygenes inheritance model in the software package SEA-G4F_2_ [38], was used to perform the genetic analysis of PH for the NY–DF F_2_ population along with the two parents and the F_1_. The results showed that PH is controlled by one major gene with additive-dominant effects. The major gene heritability was 80.04%, with an additive effect of 28.73 and a dominant effect of − 3.12 (Additional file 3: Table S2). The high heritability of the major gene indicated that PH in *df59* was relatively stable and not greatly influenced by environment; therefore, it can be selected in the early generations of a breeding process.

### QTL-seq of the NY–DF population

Two contrasting DNA pools were constructed from 16 extremely tall lines (T-pool) and 24 extremely dwarf lines (D-pool) in NY–DF F_2_ population. Illumina high-throughput sequencing generated a total of 92.986 Gb clean data for the two pools and two parental lines, with average Q20 ≥ 95.66% and Q30 ≥ 93.14%. When the obtained reads were matched to the *B. napus* “Darmor-*bzh*” reference genome, the results showed that the depths of sequencing coverage for T-pool, D-pool, NY18 and *df59* were 35.85-fold, 31.27-fold, 16.79-fold and 17.84-fold, respectively.

**Fig. 2:**
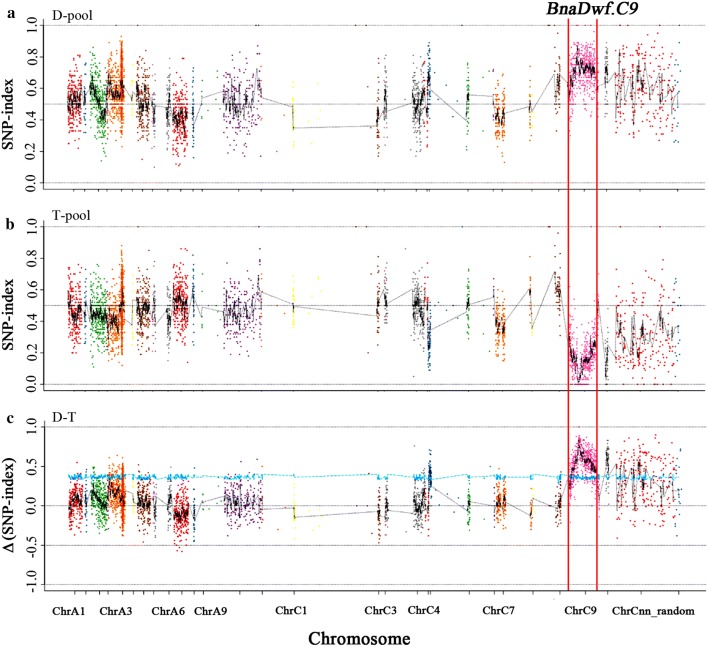
SNP-index and Δ(SNP-index) graphs from the QTL-seq analysis. SNP-indices of **a** D-pool and **b** T-pool; **c** Δ(SNP-index). The x-axes represent the *B. napus* chromosomes and the y-axes represent the SNP-index (**a**, **b**) or Δ(SNP-index) (**c**). The SNP-index and Δ(SNP-index) calculations are based on the descriptions in the “Methods”. The genomic region (17.00–27.30 Mb) on chromosome C9 had an average Δ(SNP-index) of 0.60 and is regarded as the candidate QTL, with a 95% significance level (*P* < 0.05)

Based on the genotyping, 4520 polymorphic SNPs with haplotype differences between the two parents were identified. The region (17.00–27.30 Mb) on chromosome C9 had an average SNP-index of 0.726 in the D-pool, with the highest being 1 (Fig. 2a), while the SNP-index in T-pool was 0.128, with the lowest being 0 (Fig. 2b). Then, the genome sequence of the parent NY18 was used as a reference to calculate the Δ(SNP-index) of the 4520 SNPs by combining the SNP-indices of the T- and D-pools. At a 95% significance level, the genomic region from 17.00 to 27.30 Mb had an average Δ(SNP-index) of 0.60 (Fig. 2c), suggesting that this region harbored a major QTL for PH, which was designated *BnaDwf.C9.*

### Fine-mapping the *BnaDwf.C9* locus

**Fig. 3:**
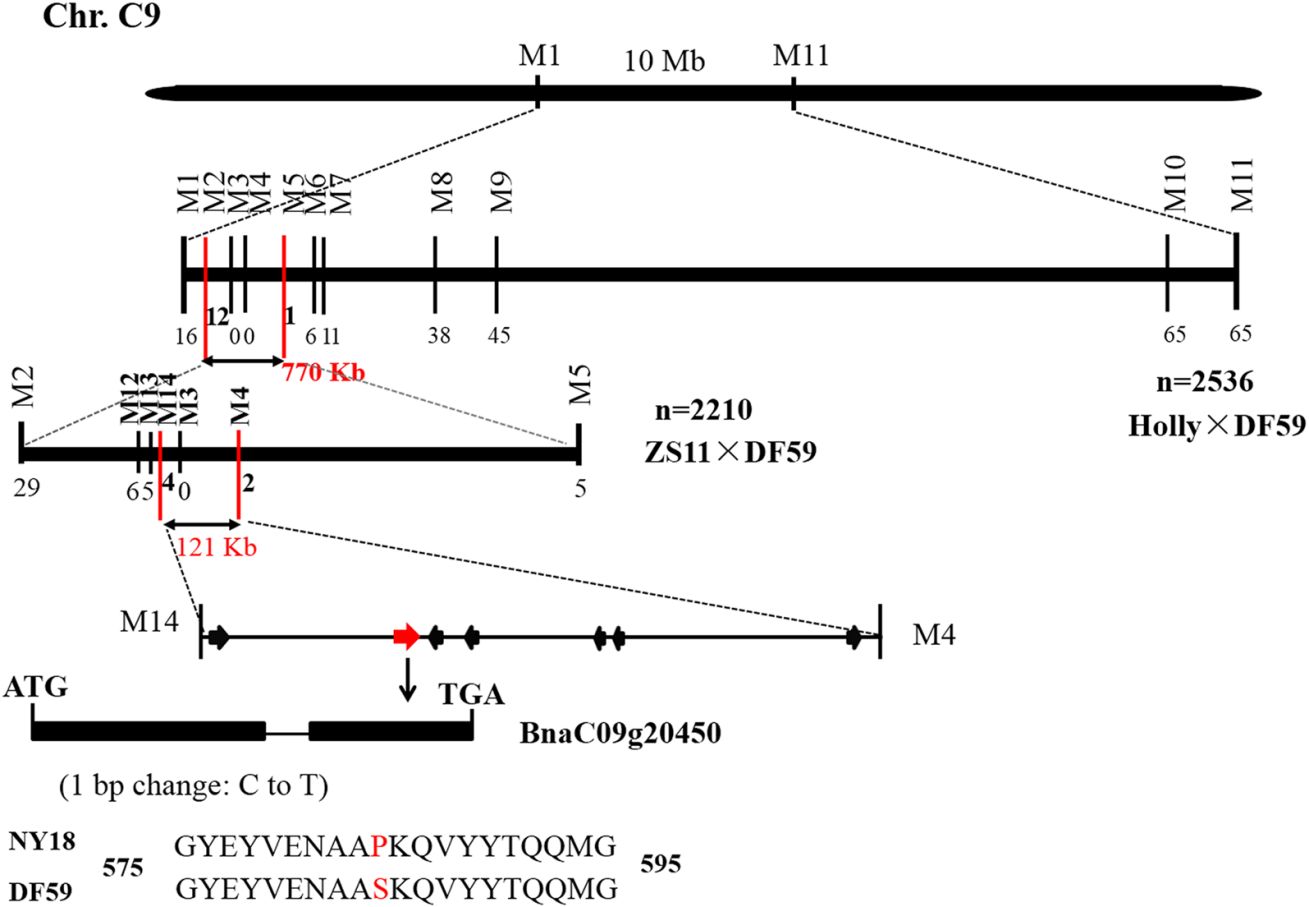
Map-based cloning and a candidate gene of *BnaDwf.C9*. First, the *BnaDwf.C9* locus was fine-mapped to a 770-kb region using the HO–DF population containing 2356 F_2_ individuals. Then, the *BnaDwf.C9* locus was further fine-mapped to the 120.87-kb region using the ZS–DF population containing 2210 F_2_ individuals. The region contains seven putative open reading frames, and only *BnaC09g20450D* had a SNP (C–T) that changed the proline at the 585th position to serine acid

To fine-map the *BnaDwf.C9* locus, two flanking penta-primer amplification refractory mutation system (PARMS) SNP markers, M1 (physical position of the *B. napus* ‘Darmor-*bzh*’ genome: 17,001,732) and M11 (27,299,112), were first designed to investigate recombinants in the F_2_ population (HO–DF) from a cross between Holly and *df59* (Additional file 4: Table S3). The results showed that 81 recombinants were obtained among the 2356 F_2_ individuals, including 56 tall recombinants and 25 dwarf recombinants (Additional file 4: Table S3). Based on the re-sequencing information of NY18 and *df59* at the target region, nine polymorphic SNP markers (M2–M10) between M1 and M11 were designed and used for genotyping the 81 recombinant individuals. Recombinant genotypes showed that M2 was located on the left side of *BnaDwf.C9*, while six SNP markers (M5–M10) were mapped on the right side (Fig. 3, Additional file 4: Table S3). In addition, M3 and M4 co-segregated with *BnaDwf.C9* and were consistent with the PH phenotype. Eventually, the location of *BnaDwf.C9* was narrowed down to the interval between M2 and M5 in a region of 770.72 kb in the ‘Darmor-*bzh*’ genome. No polymorphic SNP markers were able to narrow the interval in the HO–DF population.

For the further fine-mapping of *BnaDwf.C9*, a population of 2210 F_2_ individuals was constructed from a cross between Zhongshuang 11 (ZS11) and *df59* (ZS–DF population). Using the methods described above, 34 recombinant individuals were detected between M2 and M5 in the ZS–DF population (Table 2). Subsequently, three new polymorphic SNP markers in this region (M12–M14) were designed, together with co-segregating SNP markers (M3 and M4) in the HO–DF population and used to screen the 34 recombinants (Additional file 5: Table S4), resulting in 6, 5, 4, 0 and 2 recombinants, respectively (Fig. 3, Table 2). These results suggested that the locus *BnaDwf.C9* was fine-mapped to the region between M14 and M4, corresponding a 120.87 kb interval in the *B. napus* ‘Darmor-*bzh*’ genome (Fig. 3).

**Table 2:** Thirty-four recombinants and their genotypes detected in ZS–DF F_2_ population

Marker name	M2	M12	M13	M14	M3	M4	M5
SNP position	17,233,664	17,396,076	17,409,616	17,420,876	17,463,666	17,541,746	18,004,384
Recombinants number	29	6	5	4	0	2	5
ZS11	A	A	A	A	A	A	A
*df59*	B	B	B	B	B	B	B
F_1_	H	H	H	H	H	H	H
Dwarf individuals	H	B	B	B	B	B	B
	B	B	B	B	B	B	H
	H	H	H	H	B	B	B
	H	B	B	B	B	B	B
	B	B	B	B	B	B	H
	H	B	B	B	B	B	B
	H	B	B	B	B	B	B
	B	B	B	B	B	B	H
	H	B	B	B	B	B	B
	H	B	B	B	B	B	B
	H	B	B	B	B	B	B
	H	H	H	H	B	B	B
	B	B	B	B	B	H	H
Tall individuals	H	A	A	A	A	A	A
	H	A	A	A	A	A	A
	H	A	A	A	A	A	A
	H	A	A	A	A	A	A
	H	A	A	A	A	A	A
	H	A	A	A	A	A	A
	H	A	A	A	A	A	A
Middle individuals	B	B	H	H	H	H	H
	A	H	H	H	H	H	H
	A	H	H	H	H	H	H
	B	H	H	H	H	H	H
	H	H	H	H	H	A	A
	B	H	H	H	H	H	H
	A	H	H	H	H	H	H
	B	H	H	H	H	H	H
	A	A	A	H	H	N	H
	A	H	N	H	H	H	H
	A	A	A	A	H	H	H
	B	H	N	H	H	H	H
	A	H	H	H	H	H	H
	A	A	A	A	H	H	H

*A* genotype of tall plants, *B* genotype of dwarf plants, *H* genotype of middle plants

### Genome-wide transcriptomic analyses of NY18 and *df59*

High-throughput RNA sequencing (RNA-seq) generated 20.32‒26.83 million raw reads for each sample. After trimming the low-quality sequences, 16.29–22.37 million clean reads were aligned to the *B. napus* ‘Darmor-*bzh*’ genome (Additional file 6: Table S5). A total of 3995 differentially expressed genes (DEGs) were identified between NY18 and *df59* stem tip transcriptomes using the threshold false discovery rate < 0.005 and at least a 2.0-fold expression changed. In total, 1266 genes were significantly upregulated and 2729 genes were significantly down-regulated in *df59* compared with NY18 (Additional file 7: Table S6).

To understand gene functions associated with the dwarf phenotype in *df59*, a Gene ontology (GO) enrichment analysis of the DEGs was performed. For both up- and down-regulated genes, the most three significantly enriched GO terms in the ‘biological process’, ‘cellular component’ and ‘molecular function’ groups were the same (Additional file 8: Table S7). For example, “metabolic process”, “cellular process” and “single-organism process” were the three most enriched GO terms of the “biological process” group.

**Fig. 4:**
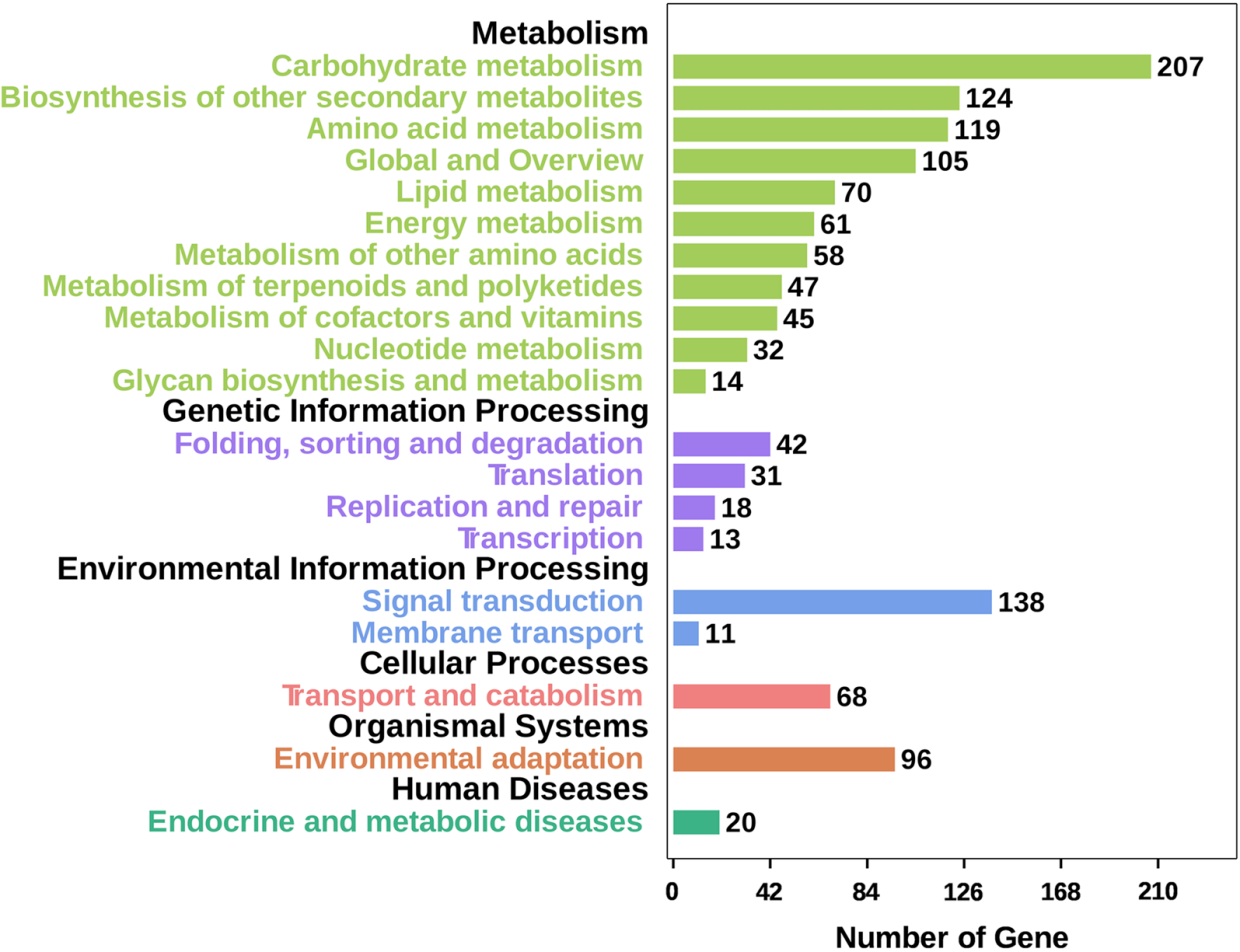
KEGG pathway categories of differentially expressed genes between NY18 and *df59* at the stem elongation stage

To discern DEG functions, we also conducted a Kyoto Encyclopedia of Genes and Genomes (KEGG) enrichment analysis. The 1266 up- and 2729 down-regulated genes were enriched in 92 and 114 pathways, respectively (Additional file 9: Table S8). The top 20 enriched pathways for all of the up- and down-regulated DEGs are shown in Fig. 4. The five most significantly enriched pathways were carbohydrate metabolism, signal transduction, biosynthesis of other secondary metabolites, amino acid metabolism, and global and overview (Fig. 4). As the PHs in *A. thaliana* and rice were mainly regulated by plant hormones [7–11], we focused on the DEGs involved in signal transduction pathway (138 genes). Among them, 118 DEGs were clustered in plant hormone-related signal transduction pathways. In total, 81 DEGs were enriched in auxin signal transduction, accounting for 68.6% of the 118 DEGs (Additional file 10: Table S9). The remaining 37 DEGs were enriched in brassinosteroid (10 genes), abscisic acid (seven genes), GA (five genes), ethylene (five genes), jasmonic acid (four genes), salicylic acid (four genes) and cytokinin (two genes) signaling pathways (Additional file 10: Table S9). This result strongly indicated that the mutation leading to the dwarf phenotype of *df59* may occur in an important gene in the auxin signal transduction. The KEGG pathway enrichment analysis provided an important clue for identifying candidate genes.

### Identification of a candidate gene

Candidate genes underlying the 17.42–17.54 Mb of *BnaDwf.C9* were analyzed based on the *B. napus* “Darmor-*bzh*” reference genome [22]. The segment harboring the *BnaDwf.C9* locus contained seven annotated or predicted genes (Table 3). An RNA-seq analysis demonstrated that none of the expression levels among the seven genes significantly differed between NY18 and *df59* at the stem elongation stage (Additional file 11: Table S10). Interestingly, *BnaC09g20480D*, *BnaC09g20490D* and *BnaC09g20500D* were not expressed at all in either NY18 or *df59*. Three other genes, *BnaC09g20440D*, *BnaC09g20460D* and *BnaC09g20470D*, showed no sequence differences between NY18 and *df59* in the open reading frames (ORFs) based on the re-sequencing results. To confirm this result, the ORFs of the three genes in NY18 and *df59* were amplified. DNA sequencing also revealed that the ORFs in NY18 and *df59* were absolutely accordant. Therefore, *BnaC09g20440D*, *BnaC09g20460D* and *BnaC09g20470D* were not candidate genes as there were neither sequence differences in the ORFs nor expression differences between NY18 and *df59*.

**Table 3:** Genes on the mapped 120.87 kb interval of “Darmor-*bzh*” reference genome and their annotation

Gene of *B. napus*	Chromosome position	Orthologous gene of *A. thaliana*	Gene function
*BnaC09g20440D*	17,421,172–17,423,263	AT2G01180	Phosphatidic acid phosphatase 1
*BnaC09g20450D*	17,461,819–17,464,062	AT2G01190	Octicosapeptide/Phox/Bem1p family protein
*BnaC09g20460D*	17,464,702–17,466,229	AT2G01250	Ribosomal protein L30/L7 family protein
*BnaC09g20470D*	17,469,391–17,470,034	AT2G01300	Na
*BnaC09g20480D*	17,481,324–17,484,464	AT2G01330	Nucleotide binding
*BnaC09g20490D*	17,493,553–17,493,792	AT1G18370	ATP binding microtubule motor family protein
*BnaC09g20500D*	17,534,536–17,535,744	AT2G01340	Na

In addition, “Ningyou 7” [39] and “ZS11” [40] reference genomes were used for the candidate gene analysis within the 120.87-kb interval harboring *BnaDwf.C9*. A new gene (*chrC09g002459*) in the “Ningyou 7” genome that was not in “Darmor-*bzh*” was identified (Additional file 11: Table S10). However, the gene was not expressed in either NY18 or *df59*. In the “ZS11” genome, *BnaC09G0251200ZS* was a new gene with no expression, while *BnaC09G0251600ZS* was a new predicted gene with a sequence length of only 240 bp, which showed neither a sequence nor expression difference between NY18 and *df59* (Additional file 11: Table S10). Therefore, these genes were excluded as candidate genes for *BnaDwf.C9*.

*BnaC09g20450D*, named *chrC09g002455* in “Ningyou 7” and divided into *BnaC09G0251300ZS* and *BnaC09G0251400ZS* in “ZS11”, is homologous to *AT2G01190* in *Arabidopsis*. *AT2G01190* belongs to the octicosapeptide/Phox/Bem1p family of proteins and encodes a protein of unknown function (Table 3). The PB1 domain (aa 69–167) is an important functional domain in *BnaC09g20450D* (Additional file 12: Figure S2). In plants, PB1-mediates interactions of ARF and Aux/IAA to modulate auxin-regulated gene transcription [41, 42]. The KEGG pathway enrichment analysis showed that most of the DEGs related to signal transduction were enriched in the auxin signal transduction pathway. The RNA-seq analysis revealed that *BnaC09g20450D* had similar transcript levels between NY18 and *df59* (Additional file 11: Table S10), while the re-sequencing analysis revealed a SNP in NY18 and *df59* (Fig. 3). Subsequently, we amplified and sequenced the promoter region (2.0-kb upstream of the start codon) and the ORF of *BnaC09g20450D* from NY18 and *df59*. No polymorphism was identified in the promoter sequences. The ORF is 1887-bp in length, encoding a protein of 628 amino acids, and a single nucleotide substitution (C to T) was identified in the second exon, which changed the proline at the 585th position to serine acid (P585S) (Fig. 3). However, the SNP did not occur in PB1 domain or any other domain (Additional file 12: Figure S2). The SNP in *BnaC09g20450D* was also developed as PARMS SNP marker M3, which co-segregated with *BnaDwf.C9* in 2536 individuals of the HO–DF population and in 2210 individuals of the ZS–DF population (Fig. 3). Therefore, we speculated that *BnaC09g20450D* was the most likely candidate gene of *BnaDwf.C9.*

### Development of a PCR-based SNP marker specific for *BnaDwf.C9*

**Fig. 5:**
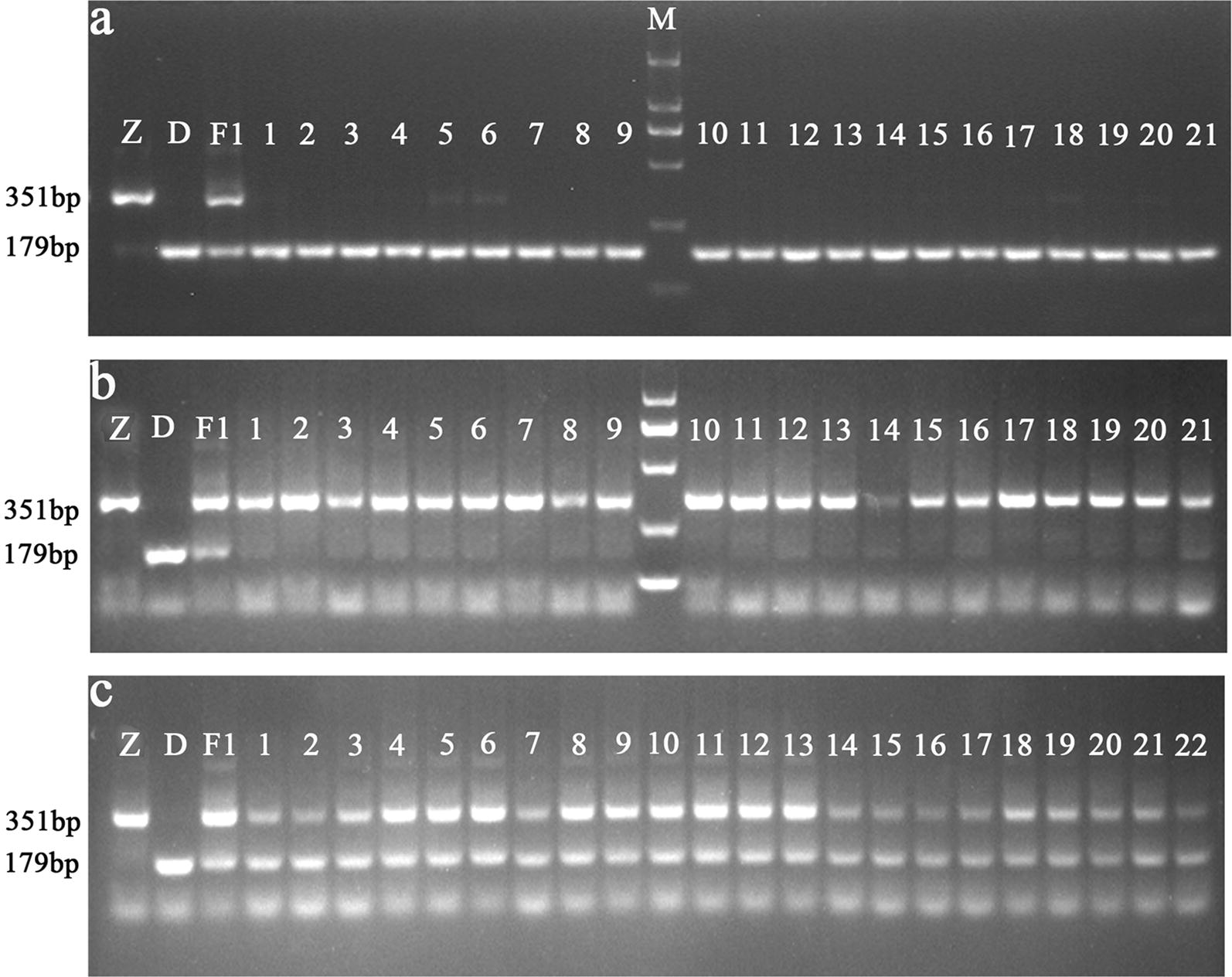
Electrophoretic profiles obtained for individuals with different plant heights from the developed SNP marker. Z: Zhongshuang 11; D: *df59*; F_1_: the F_1_ of a cross between Zhongshuang 11 and *df59*; M: DNA marker. The SNP marker can amplify **a** specific PCR products in *df59* and dwarf individuals (179-bp fragment), **b** Zhongshuang 11 and tall individuals (351-bp fragment), and **c** F_1_ and middle individuals (both 351-bp and 179-bp fragments). PCR products were analyzed by electrophoresis in 2.5% agarose gel

The SNP in the candidate gene *BnaC09g20450D* was targeted to develop a molecular marker. A SNP marker named BnaPHC9-SNP containing four primers (BnaM3pcr-F/BnaM3pcr-R/BnaM3pcr-Fc/BnaM3pcr-Rt) was designed based on the 400-bp flanking sequence of the SNP. BnaPHC9-SNP was first used to amplify NY18, *df59* and their F_1_, which produced a 351-bp fragment in NY18, a 179-bp fragment in *df59*, and both 351-bp and 179-bp fragments in the F_1_. Subsequently, 21 (tall PH), 21 (dwarfism PH) and 22 (medium PH) individuals were randomly selected from the ZS–DF F_2_ population to test the SNP marker amplification. The results of the agarose gel electrophoresis showed that only a 179-bp PCR fragment was present in individuals with a dwarf PH (Fig. 5a), only a 351-bp fragment was amplified in individuals with a tall PH (Fig. 5b), and both 179-bp and 351-bp fragments were produced in individuals with a medium PH (Fig. 5c). These results suggested that BnaPHC9-SNP could produce specific PCR amplicons from different alleles of the SNP and that it can be used for the rapid identification of the *BnaDwf.C9* locus, which confers the dwarfing trait, in breeding programs.

## Discussion

### *df59* is an elite genetic resource for semi-dwarf breeding

In the 1960s, the introduction of dwarfing traits into wheat and rice, combined with the application of improved cultivation methods, led to spectacular increases in grain yields, the so-called “green revolution” [12, 13]. *B. napus* is the third most important oilseed crop, providing 13–16% of vegetable oil globally [43], and rapeseed oil has a strong potential for use in biodiesel production [44]. In China, up to 70% of the total rapeseed cultivation areas were planted with hybrid rapeseed, because they normally produce a 25% greater seed yield and have greater yield stability [37]. However, the utilization of heterosis in rapeseed also led to the PH increasing significantly, resulting in an increased risk of lodging and a decrease in the harvest index. Owing to the lack of excellent dwarf *B. napus* germplasm resources, there is no high-yielding semi-dwarf variety widely planted, as there is for wheat and rice. *df59* is a dwarf mutant obtained from a large-scale screening of EMS-mutagenized NY18. The PH of F_1_ individuals of a cross between *df59* and NY18 was 126.75 ± 4.3 cm (Table 1), and the seed yield was 84% that of NY18, suggesting that the F_1_ are suitable for high-density planting and mechanized harvest [37]. The average harvest index in *B. napus* of 0.28 [45, 46], was much lower than those of wheat and rice (0.4–0.6) [45]. The harvest index of F_1_ individuals was 0.41, which was significantly greater than that of NY18 (0.34), indicating that the F_1_ more efficiently utilized water and soil resources (Table 1). In addition, the PH of ZS11 was 162.6 ± 5.5 cm, while the F_1_ PH of a ZS11 × *df59* cross was 114.4 ± 3.9 cm (data not shown), suggesting that allele in *df59* showed a dwarfing effect in multiple genetic backgrounds. In summary, *df59* is an outstanding male parent for breeding new semi-dwarf hybrid varieties that can be densely planted and machine harvested.

### *BnaDwf.C9* is a new locus associated with plant height in *B. napus*

In *B. napus*, hundreds of QTLs associated with PH have been identified and located on all 19 chromosomes [23–31]. Several QTLs on the A3, A6, A9, C5 and C7 chromosomes have been fine-mapped or cloned [21, 32–36]. However, only a few QTLs with minor PVs were obtained on the C9 chromosome in previous studies. For example, Shi et al. detected a minor QTL with a PV range of 3.2–4.3% [23]; Ding et al. identified a environment-specific QTL with a PV of 10.9% [25]; and Wang et al. detected four QTLs at the mature stage, with a PV range of 3.69–9.87% [26]. Using a population containing 520 diverse rapeseed accessions, Sun et al. identified 68 loci, which were distributed over the chromosomes, except for the C8 and C9 chromosomes, significantly associated with PH using a GWAS [29]. Luo et al. and Li et al. also performed GWASs for PH, and no locus significantly associated with PH was identified on the C9 chromosome [27, 28]. Thus, it appeared that there was no major QTL controlling PH on the C9 chromosome in *B. napus*. In the present study, *BnaDwf.C9* was fine-mapped to a 120.87-kb region on the C9 chromosome (Fig. 3). Although it difficult to directly compare *BnaDwf.C9* with those reported QTLs owing to the lack of common markers, we still believe that *BnaDwf.C9* is a potentially new locus, because it showed an obvious effect on PH.

### The candidate gene may regulate plant height of *df59* through the auxin-signaling pathway

The plant hormone auxin plays an essential role in most aspects of growth and developmental processes [18, 19, 47]. In most plants, auxin regulates the transcription of auxin-responsive genes through the well-established TIR1/AFB-Aux/IAA-ARF pathway [18, 19]. The Aux/IAAs contain four functional domains [18, 19]: Domain I is a repressor domain that contains a leucine repeat EAR motif [48]; Domain II is an internal domain that contains the degron motif GWPPV; Domains III and IV share high homology and contain C-terminal regions that form a PB1 protein–protein interaction domain [49]. In *B. napus*, a G-to-A mutation in the GWPPV motif of domain II causes multiple phenotypic alterations, including a reduction in PH and branch angles [21]. Among the seven genes underlying the mapped interval of *BnaDwf.C9* (Table 3, Additional file 11: Table S10), only *BnaC09g20450D* has a SNP (C-to-T mutation) between NY18 and *df59*. *BnaC09g20450D* also contains a PB1 domain (aa 69–167) (Additional file 12: Figure S2); however, the P585S substitution did not occurred in the PB1 domain or any other domain. The PB1 domain can facilitate the formation of ARF-ARF, ARF-Aux/IAA, and Aux/IAA-Aux/IAA homo- and hetero-oligomers, because the ARF and Aux/IAA proteins contain similar PB1 domains [49]. We speculate that the P585S substitution in *BnaC09g20450D* disrupts the normal function of the PB1 domain in an unknown way and then affects auxin signaling resulting in the dwarf stature of *df59*. The RNA-seq analysis also showed 81 DEGs in the auxin-signaling pathway between NY18 and *df59* (Additional file 10: Table S9). Combining the results of fine-mapping and RNA-seq, we hypothesized that the candidate gene of *BnaDwf.C9* regulated PH through the auxin-signaling pathway, but the molecular mechanism remains unknown and requires further study.

### The developed SNP marker can facilitate the application of *BnaDwf.C9* in breeding

Traditional breeding, which largely depends on the breeder’s experience and subjective judgment, is a relatively resource- and time-consuming process. Marker-assisted breeding is an effective and accurate approach to perform target-trait selection in breeding, because traits can be examined at any developmental stage, providing results without incurring environmental impacts [50, 51]. In the present study, we determined that the dwarf *B. napus* mutant *df59* has important potential application value in breeding. However, it is important to determine how to efficiently and extensively use the dwarfing gene in semi-dwarf breeding. To address this question, a molecular marker BnaPHC9-SNP was developed for *BnaDwf.C9* based on a SNP that co-segregated in two populations, containing 4746 individuals in total (Table 2, Additional file 4: Table S3). BnaPHC9-SNP was a dominant, allele-specific functional marker, which could amplify specific PCR products in *df59* (179-bp fragment), NY18 (351-bp fragment) and their F_1_ (both 351-bp and 179-bp fragments). The marker was subsequently confirmed using individuals with different PHs in the ZS–DF F_2_ population (Fig. 5), suggesting that BnaPHC9-SNP can be used to select the target PH without morphological characterization, which would speed up the breeding process. To date, a variety of high-throughput SNP genotyping methods has been developed, including the TaqMan system [52] and Kompetitive allele-specific PCR. However, for most breeders, the equipment needed in these methods is prohibitory and more expensive than using normal primers. The SNP marker developed in this study requires no fluorescence-tagged probes or real-time PCR instruments, and PCR products can be correctly detected using agarose gel electrophoresis. Therefore, BnaPHC9-SNP can be widely used in marker-assisted selection of *BnaDwf.C9* and speed up the breeding process.

## Conclusions

*Brassica napus* provides not only edible vegetable oil for human consumption, but also a triglyceride source for biofuel and lubricant production. In the present study, we isolated the dwarf mutant *df59* from an EMS-mutagenized NY18. The main agronomic traits in NY18, *df59* and their F_1_ showed that *df59* is an elite genetic resource for semi-dwarf breeding. Subsequently, the combination of QTL-seq and fine-mapping revealed a candidate gene located within an interval of 120.87 kb on the C9 chromosome. The transcriptome analysis suggested that the PH of *df59* was most likely influenced by a gene involved in auxin signal transduction. In addition, a comprehensive analysis revealed that *BnaC09g20450D* was the most likely candidate gene. Then, a molecular marker was developed based on the SNP in *BnaC09g20450D*. These results enrich our knowledge of the genetic architecture underlying PH in *B. napus* and also provide valuable resources for semi-dwarf breeding.

## Methods

### Plant materials

The dwarf mutant *df59* was isolated from EMS-mutagenized lines of NY18. NY18 is a variety cultivated by Jiangsu Academy of Agricultural Sciences, China. Mature seeds of NY18 were mutagenized with 1.0% EMS solution (W/V, Sigma-Aldrich) at pH 7.0 phosphate buffer for 12 h at 25 °C, according to the descriptions of Li et al. with modifications [53]. The mutagenized seeds (M_1_ generation) were rinsed with water for 4 h and sown in the field. Individual plants were bagged at the flowering stage. Self-pollination seeds of approximately 10,000 individual plants were harvested. Each M_2_ seeds were then sown into independent line. Dwarf lines were bagged to harvest seeds for evaluation in the M_3_ generation.

The NY–DF F_2_ population containing 165 individual lines derived from a cross between NY18 and *df59* was used for genetic inheritance and QTL-seq. Two conventional rapeseed cultivars, Holly and ZS11, were used as parental lines to develop segregating populations for fine-mapping of the QTLs associated with dwarf architecture. Holly is a Canadian spring variety, while ZS11 is an elite Chinese semi-winter rapeseed cultivar. The HO–DF F_2_ population derived from a cross between Holly and *df59* contained 2536 lines, while the ZS–DF F_2_ population derived from a cross between ZS11 and *df59* contained 2210 lines.

### Trait measurement

NY–DF, HO–DF and ZS–DF populations, as well as their parents and the F_1_ were all planted in the field of Jiangsu Academy of Agricultural Sciences, Nanjing, Jiangsu Province, China. No specific permissions were required for the field trials. The field experiments were conducted in accordance with Wang et al. with 20 plants per row and 40 cm between the rows [54]. The PH values for all the materials were measured at the mature stage.

At maturity, five open-pollinated plants each of NY18, *df59* and their F_1_ growing in the middle of the plot were selected. Agronomic traits and seed yield-related traits were measured in accordance with the description of Zhao et al. [55] and Wang et al. [56], including PH, biomass yield, seed yield, thousand seed weight, first effective branch height, first effective branch number, internode length, harvest index, length of main inflorescence, pod number of main inflorescence and silique number per plant. The harvest index was calculated as the ratio of seed yield to biomass yield [45]. In total, 10 well-developed siliques, which were randomly selected from the first branch adjacent to the main inflorescence, were used to determine the silique-related traits of NY18, *df59* and their F_1_, including silique length, silique breadth, seed number per silique and silique volume [56]. The seed oil content was measured by nuclear magnetic resonance using standard methods and fatty acid compositions were determined by near infrared reflectance spectroscopy in accordance with Chen et al. [57].

To investigate root traits at the seeding stage, four plump seeds each of NY18, *df59* and the F_1_ were planted in a seed germination pouch (CYG-19LB; Pheno Trait Technology, Co., Ltd, Beijing, China) at 25 °C, and three repetitions were conducted. After 10 days, total root length (cm), root surface area (cm^2^), root volume (cm^3^) and number of root tips for each seeding were measured using LA-S Root Analysis software (Wanshen Ltd, Hangzhou, China).

### Statistical and QTL-seq analyses

The mixed major-gene plus polygenes inheritance model of the software package SEA-G4F_2_ was used to identify the inheritance of PH in the NY–DF F_2_ population [38].

From the 165 lines of the NY–DF F_2_ population, 16 extremely tall lines and 24 extremely dwarf lines were selected. Genomic DNA was extracted from young leaves using a Plant Genomic DNA Kit (Tiangen, Beijing, China), and T-pool and D-pool were constructed by mixing equal ratios of appropriate individual DNAs. Sequencing libraries of the two bulks and two parents were constructed, and sequence data were generated using the Illumina HiSeq™ PE150 (Illumina, Inc; San Diego, CA, USA) platform. Both data sequencing and data analyses were performed by Novogene Bioinformatics Technology Co. Ltd. (Beijing, China). The raw data were filtered through a series of quality controls which resulted in the removal of reads with ≥ 10% unidentified nucleotides, > 50% bases having phred quality < 5 or > 10 nt aligned to the adapter. The clean reads of each sample were aligned against the *B. napus* “Darmor-*bzh*” reference genome [22] using BWA software [58], and the SAMtools command “rmdup” was used to remove multiple read pairs [59]. Variant calling was performed for all the samples using the Unified Genotyper function in GATK software [60]. SNPs were determined using the Variant Filtration parameter in GATK. Using NY18 as the reference parent, the above two bulks SNP-indices were calculated as the proportion of reads containing SNPs not found in NY18. The Δ(SNP-index) was calculated using the formula: Δ(SNP-index) = SNP-index (D-pool) − SNP-index (T-pool). A QTL was considered a candidate associated with PH if the Δ(SNP-index) was significantly different (*P *< 0.05).

### Fine-mapping of the QTL for plant height

The major QTL for PH was identified based on QTL-seq and named *BnaDwf.C9*, and SNPs underlying the confidence interval of *BnaDwf.C9* were obtained. Total DNAs of HO–DF and ZS–DF F_2_ individuals were extracted from fresh leaves using a modified cetyl-trimethylammonium bromide method [61]. PARMS was used to screen recombinant plants for fine-mapping among HO–DF and ZS–DF F_2_ populations. The principle of PARMS SNP genotyping is similar to that of Kompetitive allele-specific PCR assays [62]. The master mix for PARMS markers was purchased from Gentides Biotech Co., Ltd. (Wuhan, China). Detailed information for conducting the qPCR-based PARMS assay was previously published by Zhang et al. [62] and Liu et al. [63].

PARMS SNP markers flanking the confidence interval of *BnaDwf.C9* were screened from the two parents, the F_1_, and 10 individuals each of the extremely tall and dwarf lines of the HO–DF population. Then two polymorphic markers (M1 and M11) were used to screen the 2536 individuals of the HO–DF F_2_ population (Fig. 3). Combined with phenotypes, plants containing recombinants between the two markers were selected. Furthermore, the recombinant plants were analyzed with newly developed polymorphic PARMS SNP markers (M2–M10), and *BnaDwf.C9* was finally mapped to an interval between M2 and M5 (Fig. 3).

For the further fine-mapping of *BnaDwf.C9*, M2 and M5 were used to screen the 2210 individuals of the ZS–DF F_2_ population. Using the same method as described above, plants containing recombinants between M2 and M5 were selected. Three new polymorphic PARMS SNP markers (M13–M15) together with co-segregating SNP markers (M3 and M4), were used to analyze the selected recombinant plants (Fig. 3, Additional file 5: Table S4). Finally, we narrowed down *BnaDwf.C9* to a genomic region between the markers M14 and M4.

### RNA library construction and sequencing

Equivalent amounts of stem tips from NY18 and *df59* at the stem elongation stage were collected for RNA extraction, and two biological replicates were performed. Total RNA was extracted using a MiniBEST Plant RNA Extraction Kit (TaKaRa, Dalian, China). RNA-seq and data analysis were carried out by Novogene Bioinformatics Technology Co. Ltd. In brief, the RNA concentration was measured using a Qubit^®^ RNA Assay Kit and a Qubit^®^ 2.0 Fluorometer (Life Technologies, Carlsbad, CA, USA). The RNA integrity was assessed using an RNA Nano 6000 Assay Kit and the Bioanalyzer 2100 system (Agilent Technologies, Santa Clara, CA, USA). Each of the two sequencing libraries for NY18 and *df59* were constructed using an NEBNext^®^ Ultra™ RNA Library Prep Kit for Illumina^®^ (NEB, USA) following the manufacturer’s instructions, and the library quality was assessed on the Agilent Bioanalyzer 2100 system. The four libraries were sequenced on an Illumina HiSeq 2000 platform, and 100 bp paired-end reads were generated. These methods were described by Yu et al. with modifications [64].

### RNA-seq data analysis

Raw RNA-seq reads were processed to remove reads containing the adapter, reads containing ploy-Ns and low-quality reads. Paired-end clean reads were aligned to the *B. napus* “Darmor-*bzh*” reference genome [22] using TopHat version 2.0.6 [65]. The read numbers mapped to each gene were counted using HTSeq version 0.6.1 [66], and reads per kilobase of exon per million reads of each gene were calculated. Differential expression analyses of NY18 and *df59* (two biological replicates per sample) were performed using the DESeq R package (1.10.1). Genes with a false discovery rate < 0.005 and log_2_(fold change) ≥ 1 were declared DEGs.

The GO annotation of DEGs was performed using the GOseq R package [67], and GO terms with corrected *P* < 0.05 were considered to be significantly enriched terms. In addition, DEGs were submitted to the KEGG (https://www.genome.jp/kegg/) website, and KEGG enrichment pathways of DEGs were determined using KOBAS online analysis database [68].

### Development of an allele-specific marker for traditional PCR amplification

Based on the results of the fine-mapped *BnaDwf.C9*, the PARMS SNP marker M3 co-segregated with the *BnaDwf.C9* gene associated with PH. To confirm this SNP, the coding region of the gene that contained the SNP was amplified and sequenced from NY18 and *df59*. A region, including 200 bp both upstream and downstream of the SNP, was considered the target region. A SNP marker containing four primers and named BnaPHC9-SNP was designed for the allele-specific amplification of the SNP. The primers were a forward locus primer (BnaM3pcr-F: 5′-GAGAAATACTCCGCAACCTACG-3′), reverse locus primer (BnaM3pcr-R: 5′-ATGTTCCGAAACCAACCAGAG-3′), allele primer 1 (BnaM3pcr-Fc: 5′-TATGAATATGTGGAAAATGAGC-3′) and allele primer 2 (BnaM3pcr-Rt: 5′-GCGTGTAGTATACCTGCTTGGA-3′). BnaM3pcr-F began amplifying from 157-bp upstream of the SNP, while BnaM3pcr-R began amplifying from 193-bp downstream of the SNP. BnaM3pcr-Fc and BnaM3pcr-Rt were upstream and downstream, respectively, with 3′-terminal bases of C and A (according to the allele of NY18 or *df59*), respectively. A mismatch base was introduced at the third-to-last base of the primer BnaM3pcr-Fc. All the primer oligonucleotides were synthesized by Tsingke Biological Technology Co., Ltd. (Wuhan, China).

The PCR mixture (including dNTPs, Taq buffer and Taq enzyme) was purchased from Gentides Biotech Co., Ltd. The PCR reagent mixture (20 μL total volume) contained: 2 × PCR MIX: 10 μL, BnaM3pcr-F: 0.8 μL, BnaM3pcr-R: 0.8 μL, BnaM3pcr-Fc: 0.8 μL, BnaM3pcr-Rt: 0.8 μL, DNA: 1 μL (50 ng/μL), ddH_2_O: 5.8 μL. The PCR assay was conducted as described by Zhang et al. with modifications [62], as follows: denaturation at 94 °C for 15 min, followed by 10 cycles of 94 °C for 20 s and 65 °C (− 0.8 °C per cycle) for 1 min, followed by 30 cycles of 94 °C for 20 s and 57 °C for 1 min, and a final extension at 72 °C for 5 min. PCR products were analyzed by electrophoresis in 2.5% agarose gel for 40 min at 100 V in TAE buffer (40 mM Tris–acetate, 1 mM EDTA, pH 8.0).

## Supplementary information

**Additional file 1: Table S1.** The phenotypic values of seed oil content and seed fatty acid concentrations for the NY18, *df59* and the F_1_.

**Additional file 2: Figure S1.** Root related traits of NY18, *df59* and their F_1_ at 10 days after germination.

**Additional file 3: Table S2.** Genetic parameters estimated in one major gene with additive-dominant model in the NY–DF F_2_ population.

**Additional file 4: Table S3.** Eighty-one recombinants and their genotypes detected in HO–DF F_2_ population.

**Additional file 5: Table S4.** PARMS SNP markers used for fine-mapping of the *BnaDwf.C9* locus in ZS–DF F_2_ population.

**Additional file 6: Table S5.** Summary of transcriptome sequencing data.

**Additional file 7: Table S6.** The differentially expressed genes between NY18 and *df59*.

**Additional file 8: Table S7.** GO terms for the differentially expressed genes between NY18 and *df59*.

**Additional file 9: Table S8.** KEGG pathways for the differentially expressed genes between NY18 and *df59*.

**Additional file 10: Table S9.** The 118 differentially expressed genes clustered in plant hormone-related signal transduction pathways.

**Additional file 11:** Genes on the mapped 120.87 kb interval of “Darmor-*bzh*”, “Ningyou 7” and “Zhongshuang 11” reference genomes and their expression pattern.

**Additional file 12: Figure S2.** The conserved domains for the candidate gene *BnaC09g20450D*.

## Data Availability

The raw sequence data have been deposited in the NCBI (https://www.ncbi.nlm.nih.gov/sra/) Sequence Read Archive (SRA) under Accession numbers SRR10915207, SRR10915208, SRR10915209 and SRR10915210. All other relevant data during this study are included in the manuscript and additional files.

## Abbreviations

ARF: Auxin response factor
Aux/IAA: Auxin/indole acetic acid protein
*B. napus*: *Brassica napus*
DEG: Differentially expressed gene
D-pool: Dwarf pool
EMS: Ethyl methanesulphonate
GA: Gibberellic acid
GO: Gene ontology
GWAS: Genome-wide association Study
KEGG: Kyoto Encyclopedia of Genes and Genomes
IAA: Indole-3-acetic acid
NY18: Ningyou 18
ORF: Open reading frames
PH: Plant height
PV: Phenotypic variance
QTL: Quantitative trait locus
SNP: Single-nucleotide polymorphism
TIR1/AFB: Transport inhibitor resistant1/auxin signaling F-box
T-pool: Tall pool
ZS11: Zhongshuang 11
