# Supplementary figures and images for: Fine-mapping and transcriptome analysis of a candidate gene controlling plant height in Brassica napus L
Source: Biotechnol Biofuels. 2020 Mar 10;13:42. doi: 10.1186/s13068-020-01687-y (PMC7063735; doi:10.1186/s13068-020-01687-y)

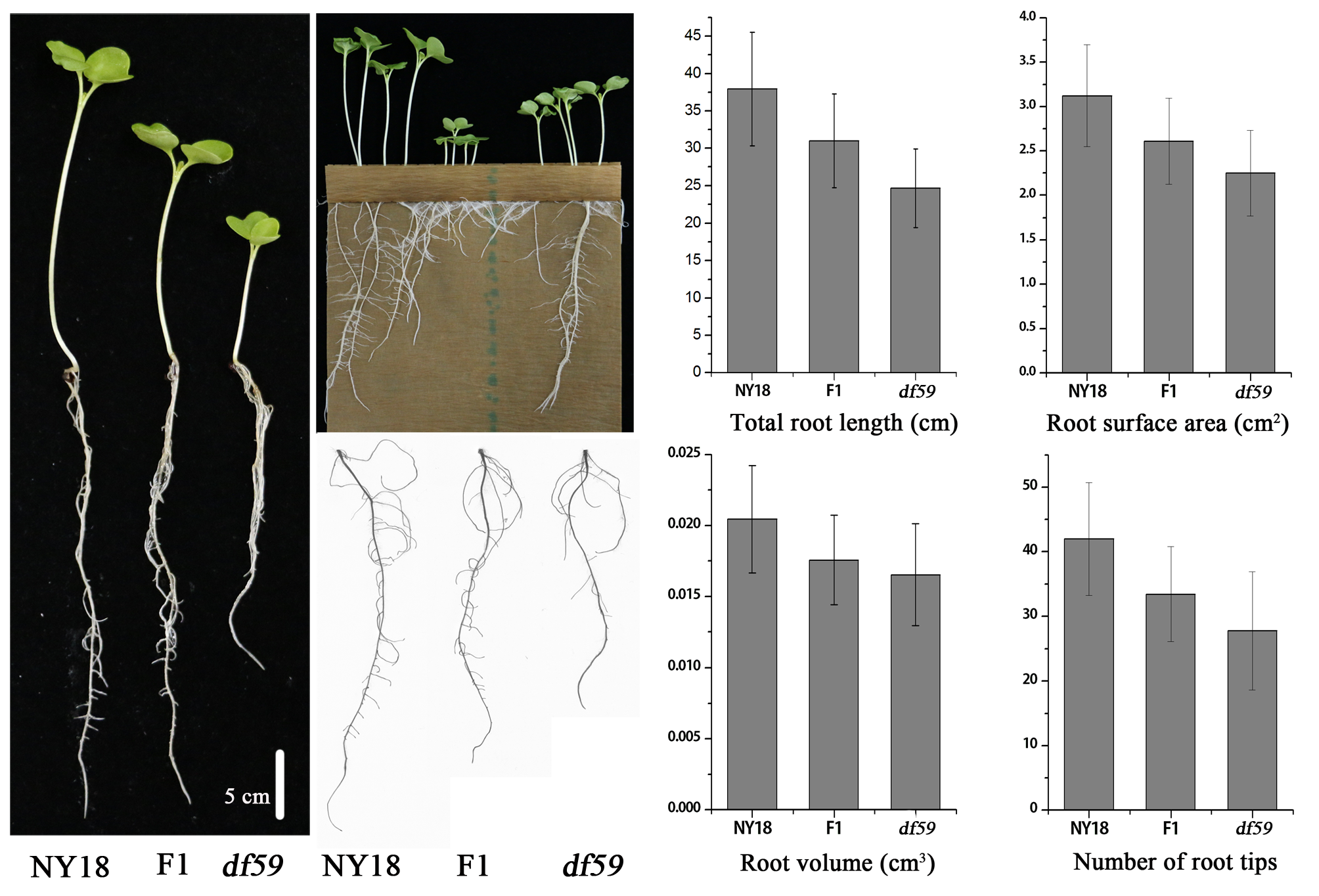

Supplement: Supplementary file 2 — Additional file 2: Figure S1. Root related traits of NY18, df59 and their F1 at 10 days after germination. [file 13068_2020_1687_MOESM2_ESM.tif]

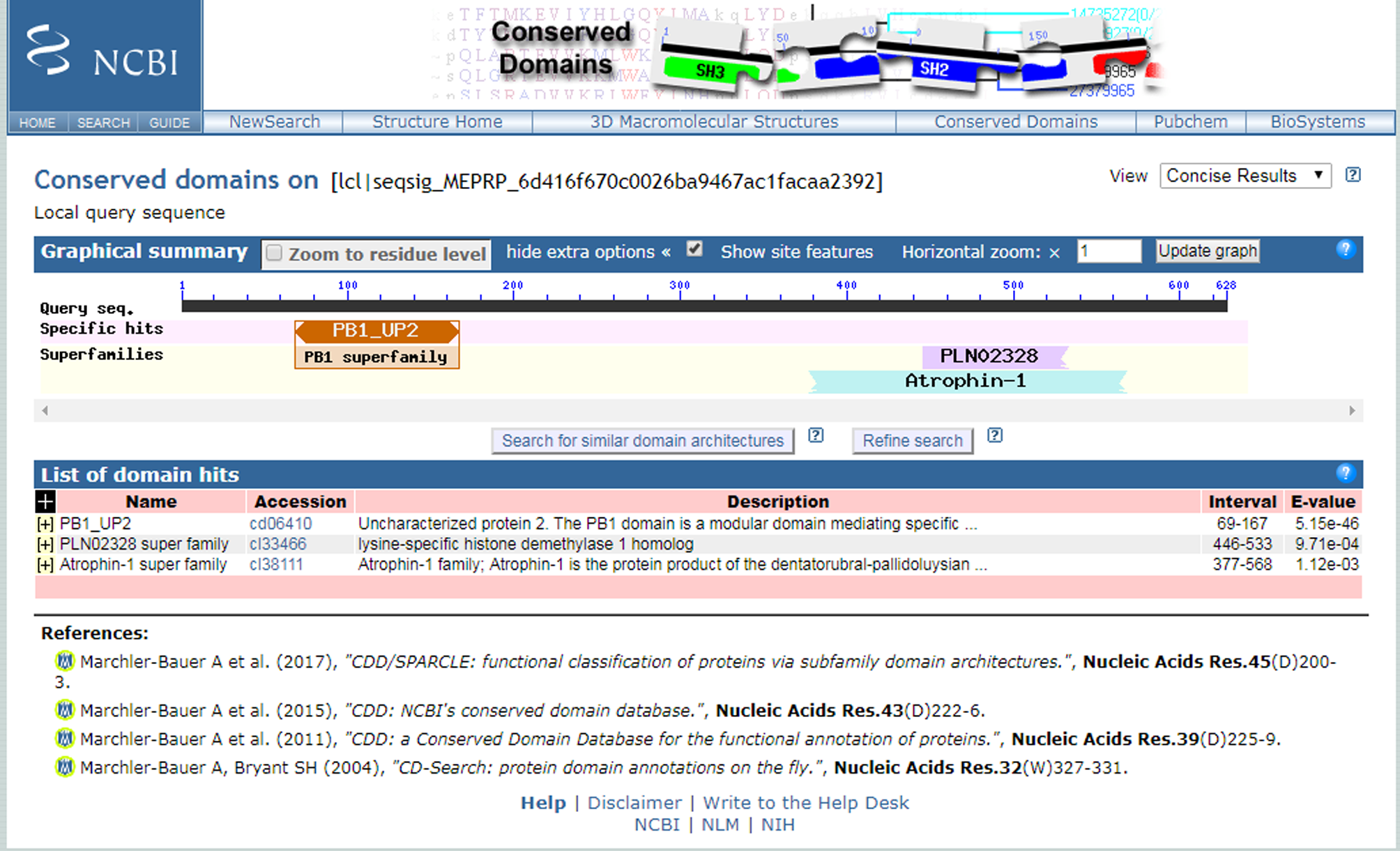

Supplement: Supplementary file 12 — Additional file 12: Figure S2. The conserved domains for the candidate gene BnaC09g20450D. [file 13068_2020_1687_MOESM12_ESM.tif]
