# Supplementary material for: Fine-mapping and transcriptome analysis of a candidate gene controlling plant height in Brassica napus L
Source: Biotechnol Biofuels. 2020 Mar 10;13:42. doi: 10.1186/s13068-020-01687-y (PMC7063735; doi:10.1186/s13068-020-01687-y)
Supplement: Supplementary file 3 — Additional file 3: Table S2. Genetic parameters estimated in one major gene with additive-dominant model in the NY–DF F2 population. [file 13068_2020_1687_MOESM3_ESM.docx]

Table S2 Genetic parameters estimated in one major gene with additive-dominant model in the NY-DF F_2_ population.

| First order | parameter | Second order | parameter |
| --- | --- | --- | --- |
| m | 86.79 | *σ*^2^_p_ | 517.03 |
| *d(da)* | 28.73 | *σ*^2^_mg_ | 413.83 |
| h(ha) | -3.12 | *σ*^2^_pg_ | 103.19 |
|  |  | *σ*^2^_e_ | 0.01 |
|  |  | *h*^2^_mg_(%） | 80.04 |

Note: *m*: Mean value; *d*: The additive effect of major gene; h: The dominant effect of major gene; *σ*^2^_p_: Population variance; *σ*^2^_mg_: Major gene variance; σ^2^_pg_: Residual and Polygene variance; *σ*^2^_e_: Environmental variance; *h*^2^_mg_(%):of Major gene Heritability; *h*^2^_pg_(%): Heritability of the polygene.
