# Supplementary material for: Fine-mapping and transcriptome analysis of a candidate gene controlling plant height in Brassica napus L
Source: Biotechnol Biofuels. 2020 Mar 10;13:42. doi: 10.1186/s13068-020-01687-y (PMC7063735; doi:10.1186/s13068-020-01687-y)
Supplement: Supplementary file 6 — Additional file 6: Table S5. Summary of transcriptome sequencing data. [file 13068_2020_1687_MOESM6_ESM.docx]

Table S5. Summary of transcriptome sequencing data.

| Sample name | Raw reads | Clean reads (Clean/All) | Q30 (%) | Total mapped (Mapped/Clean) | Uniquely mapped (Uniquely/Clean) |
| --- | --- | --- | --- | --- | --- |
| NY18-1 | 26837952 | 25641248 (94.26%) | 95.26 | 22372241 (87.25%) | 21160625 (82.53%) |
| NY18-2 | 20327320 | 18816732 (95.25%) | 92.87 | 16288577 (86.56) | 15429736 (82.00%) |
| df59-1 | 22898982 | 21585095 (95.54%) | 92.91 | 18744909 (86.84) | 17760210 (82.28%) |
| df59-2 | 23640485 | 22518336 (92.57%) | 95.22 | 19500307 (86.60) | 18450396 (81.93%) |
